# High contrast microstructural visualization of natural acellular matrices by means of phase-based x-ray tomography

**DOI:** 10.1038/srep18156

**Published:** 2015-12-14

**Authors:** Charlotte K. Hagen, Panagiotis Maghsoudlou, Giorgia Totonelli, Paul C. Diemoz, Marco Endrizzi, Luigi Rigon, Ralf-Hendrik Menk, Fulvia Arfelli, Diego Dreossi, Emmanuel Brun, Paola Coan, Alberto Bravin, Paolo De Coppi, Alessandro Olivo

**Affiliations:** 1 University College London, Department of Medical Physics and Biomedical Engineering, London, WC1E 6BT, United Kingdom; 2 University College London, Institute of Child Health, London, WC1N 1EH, United Kingdom; 3 University of Trieste, Department of Physics, Trieste, 34127, Italy; 4 Istituto Nazionale di Fisica Nucleare, Sezione di Trieste, Trieste, 34127, Italy; 5 Sincrotrone Trieste SCpA, Basovizza/Trieste, 34012, Italy; 6 European Synchrotron Radiation Facility, Grenoble, 38043, France; 7 Ludwig Maximilians University, Department of Physics, Garching, 85748, Germany; 8 Ludwig Maximilians University, Faculty of Medicine, Grosshadern-Munich, 81377, Germany

## Abstract

Acellular scaffolds obtained via decellularization are a key instrument in regenerative medicine both *per se* and to drive the development of future-generation synthetic scaffolds that could become available off-the-shelf. In this framework, imaging is key to the understanding of the scaffolds’ internal structure as well as their interaction with cells and other organs, including ideally post-implantation. Scaffolds of a wide range of intricate organs (esophagus, lung, liver and small intestine) were imaged with x-ray phase contrast computed tomography (PC-CT). Image quality was sufficiently high to visualize scaffold microarchitecture and to detect major anatomical features, such as the esophageal mucosal-submucosal separation, pulmonary alveoli and intestinal villi. These results are a long-sought step for the field of regenerative medicine; until now, histology and scanning electron microscopy have been the gold standard to study the scaffold structure. However, they are both destructive: hence, they are not suitable for imaging scaffolds prior to transplantation, and have no prospect for post-transplantation use. PC-CT, on the other hand, is non-destructive, 3D and fully quantitative. Importantly, not only do we demonstrate achievement of high image quality at two different synchrotron facilities, but also with commercial x-ray equipment, which makes the method available to any research laboratory.

The demand for organ transplantation has rapidly increased during the past decade. At the same time, a severe donor shortage and the likely rejection of transplant organs prevent this demand from being met. Tissue engineering (TE), an emerging sub-discipline of regenerative medicine, offers a therapeutic alternative. It aims at developing suitable replacements for tissues and organs from cell-free scaffolds, which are populated with stem cells of the organ recipient^1^. In general, the challenge is to generate scaffolds that allow and promote cell growth, which requires biocompatibility, an appropriate biodegradation profile, and mechanical properties, components and microarchitecture that mimic the environment of the organ to be engineered. Scaffolds have been traditionally divided into synthetic materials and natural acellular matrices (ACMs). While synthetic scaffolds have shown considerable success in both pre-clinical and clinical applications for simple organs such as the bladder, urethra and trachea^2^,^3^,^4^, so far they found limited application for the production of more complex modular organs such as the liver and the lung. Conversely, ACMs hold high potential in the production of complex scaffolds due to their intrinsic ability to recreate the three-dimensional macro- and microarchitecture of each organ^5^. As such, understanding their interactions with the cells used to repopulate them can provide a key instrument for the future development of more complex synthetic scaffolds: this requires a suitable non-destructive imaging method, which is the main topic of the paper.

ACMs are derived from animal or human organs that have been treated to remove cells and immunogenic material. An appropriate decellularization procedure removes cellular material whilst preserving tissue microarchitecture, mechanical properties, extracellular matrix (ECM) and associated growth factors. However, ACMs have been generally obtained using harsh chemicals that tend to destroy the ECM, hence hampering translation into clinical use. Current research is aimed at developing “gentle” decellularization strategies that eliminate cell population while preserving microarchitecture and ECM components^6^. This is especially important for organs that are intricate both in structure and in functionality (e.g. lung, liver, kidneys and the organs belonging to the gastrointestinal tract), as the preservation of microarchitecture is a crucial requirement for accurate cell growth and eventual transplantation. Validation of the decellularization process requires understanding whether the native tissue microarchitecture has been preserved. While this would be achieved by visualizing scaffolds after decellularization, the TE community currently lacks an imaging modality capable of producing images of sufficiently high contrast and resolution while being non-destructive^7^. Most established imaging modalities can only be used to a limited extent. MicroCT fails to achieve sufficient soft tissue contrast. Magnetic Resonance Imaging (MRI) struggles to achieve the required spatial resolution (ideally micrometres) within short scan times. Moreover, MRI scanners are costly and may be difficult to access, while a suitable imaging modality would be required to cope with a high specimen throughput. Histology and scanning electron microscopy (SEM) have provided useful images of ACMs^8^ and are the most commonly used means to validate decellularization methods; however, they both require destructive sample preparation. This is a major drawback considering that scaffolds cannot be imaged prior to transplantation. Moreover, implanted scaffolds will ultimately have to be monitored *in vivo*, which stresses the urgent requirement for non-destructive, high contrast and high resolution imaging.

In this article, we demonstrate that the limits of established imaging modalities can be overcome by x-ray phase contrast (PC) computed tomography (CT), a recently heavily investigated radiographic method that exploits phase shift rather than attenuation differences to generate contrast^9^. PC-CT has been previously applied to tissue engineering specimens^10^,^11^; however, a broad range of samples has not yet been considered. We present images of ACMs derived from esophagus, liver, lung and small intestine, as these organs are presently of major interest to the TE community^12^,^13^,^14^,^15^. Failure of these organs mostly requires transplantation as a definite treatment, and due to a critical donor shortage, they strongly rely on advances in regenerative medicine.

Scaffolds were derived via a novel, “gentle” decellularization method named detergent enzymatic treatment (DET) and variations thereof^6^,^8^,^16^. Previously, DNA quantification, histology and SEM have shown that DET reliably preserves scaffold microarchitecture. Owing to its high resolution, strong soft tissue contrast and volumetric nature, we demonstrate that PC-CT enables the same assessment without any staining or destructive sample preparation. This is a highly valuable result for the TE community, since a) it enables an easy assessment of the reliability of the decellularization process and b) it allows imaging scaffolds prior to transplantation, which means that the method could ultimately be used for quality control in regenerative medicine. Moreover, the non-destructive nature and high penetration of x-ray phase imaging methods might in the future allow for post-transplantation follow-up of transplanted scaffolds *in vivo*.

While most imaging was performed with synchrotron radiation, a key result presented in this paper is the demonstration that a comparable image quality is obtained with a laboratory-based setup, which allows a fast and widespread adoption of the method within the TE community. Although here we demonstrate image quality comparable to that obtained at synchrotrons by using an edge-illumination based PC-CT system, one could expect similar image quality also with a grating-based system^17^, making the reported results even more general and accessible to a wider community.

## Materials and Methods

### Sample preparation

All surgical procedures and animal husbandry were carried out in accordance with the UK Home Office guidelines under the Animals (Scientific Procedures) Act 1986 and were approved by the local ethics committee (University College London, UK; project license: 70/2719).

#### Esophagus

Adult New Zealand rabbits were euthanized by administration of an overdose of intravenous Pentobarbital Sodium (Sigma, UK). The abdomen was sterilized with 70 % ethanol and a midline incision was made to completely expose the abdominal and thoracic cavities. The esophagus was harvested from the cervical portion to the gastresophageal junction. The esophageal lumen was cannulated and washed with phosphate buffered saline containing 5% antibiotic antimycotic solution (PBS/AA; Sigma, UK). It was perfused with continuous fluid delivery using a Masterflex L/S variable speed roller pump (Cole-Parmer, UK) at 1 ml/min. The esophagus was infused with the detergent-enzymatic treatment (DET) consisting of deionized water (resistivity 18.2 MU/cm; dH_2_O) at 4 °C for 24 hours, 4% sodium deoxycholate (Sigma, UK; SDC) at room temperature (RT) for 4 hours, and 2000 kU deoxyribonuclease-I (Sigma, UK; DNase-I) in 1 M sodium chloride (Sigma, UK; NaCl). The process was repeated for three cycles to ensure sufficient decellularization. Following treatment, the constructs were preserved at 4 °C in PBS/AA. The samples were fixed in 2.5 % glutaraldehyde (GA) in 0.1 M phosphate buffer and left for 24 hours at 4 °C. Following GA fixation, samples were fixed in 1 % OsO_4_/0.1 M phosphate buffer (pH 7.4). After rinsing with dH_2_O, specimens were dehydrated in a graded ethanol-water series to 1000% ethanol and critical point dried using CO_2_.

#### Lung

Adult Sprague-Dawley rats, weighing 320–350 g, were euthanized by CO_2_ inhalation and exsanguination. A midline thoracotomy was performed, the muscles of the head and neck were dissected away from the midline and the trachea was sectioned above the cricoid cartilage. The thymus was removed and the pulmonary artery cannulated via the right atrium, secured with sutures, flushed with PBS/AA and the inferior and superior vena cavae were transected. The lung was mobilized from the cricoid cartilage and dissected free from its attachments to the esophagus and thoracic cavity. The trachea was cannulated and flushed with PBS/AA to wash the vascular tree and prevent coagulation. The specimens were decellularized via DET with intratracheal insufflation using either continuous or intermittent inflation. In the continuous group (“continuous DET”), the trachea was perfused using a Masterflex L/S variable speed roller pump at 0.6 ml/min. The lung was perfused with dH_2_O at 4 °C for 24 hours, 4 % SDC at RT for 4 hours, and 2000kU  DNase-I in 1M NaCl at RT for 3 hours. In the intermittent group (“Intermittent DET”), the trachea was perfused using a syringe pump. Simulating the inspiratory cycle, each insufflation of the syringe pump was followed up by a withdrawal of the liquid that was infused. Four consecutive insufflations (each lasting 30 seconds) of each solution (dH_2_O, 4 % SDC, and 2000 kU DNase-I in 1 M NaCl) comprised one cycle. Nine cycles were carried out to ensure sufficient decellularization. Following treatment, the constructs were preserved at 4 °C in PBS/AA. The samples were fixed and critical point dried in the same fashion as the esophagus samples (see previous sub-section). A non-decellularized control (“fresh”) lung specimen was prepared in addition to the decellularized ones.

#### Liver

Adult Sprague-Dawley rats, weighing 320–350 g, were euthanized by CO_2_ inhalation and cervical dislocation. The abdomen was sterilized with 70% ethanol and a U-shaped incision was performed to expose the abdomino-pelvic cavity. The abdominal inferior vena cava (IVC) and portal vein (PV) were identified and the PV was cannulated with a 24 G cannula (BD, UK), which was secured in place with a 3–0 silk suture (Ethicon, UK). The abdominal IVC was blocked using silk sutures proximal to the right renal vein and the IVC was sectioned. The diaphragm was used as a holding point to release the whole liver from the supporting tissue. The whole procedure was carried out with special caution not to damage the Glisson’s capsule, which surrounds the organ. The liver was washed with PBS/AA prior to decellularization. Specimens were decellularized with intraportal insufflation using either DET or EDTA-DET (ethylenediaminetetraacetic acid detergent enzymatic treatment). For DET treatment, the liver was connected to a Masterflex L/S variable speed roller pump and perfused with dH_2_O for 36 hours at 4 °C. Differently, for EDTA-DET treatment, the liver was connected to a Masterflex L/S variable speed roller pump and perfused with 2 mM EDTA for 15 minutes and with dH_2_O for 36 hours at 4 °C. Both DET and DET-EDTA livers were then transferred at RT and perfused with 4% SDC for 6 hours followed by perfusion of 2000 kU of DNase-I from bovine pancreas in 1M NaCl for 3 hours. The flow rate was kept at 4.5 ml/min for the dH_2_O infusion and 6.5 ml/min for SDC and DNase steps. Following the end of the decellularization cycle, the liver scaffolds were perfused with PBS/AA for 30 minutes and stored in PBS/AA at 4 °C. The samples were fixed and critical point dried in the same fashion as the previously described samples. A non-decellularized control (“fresh”) liver specimen was prepared in addition to the decellularized ones.

#### Small intestine

Adult Sprague-Dawley rats, weighing 320–350 g, were euthanized by CO_2_ inhalation and cervical dislocation. Once sacrificed, the abdomen was sterilized with 70% ethanol, and a midline incision was made to completely expose the abdominal cavity. The superior mesenteric artery (SMA) was cannulated with a 27 G cannula (BD, UK) and flushed with PBS to wash the vascular tree and prevent coagulation. The small intestine was dissected free and removed en bloc from pylorus to ileocecal valve and the intestinal lumen cannulated and washed with PBS/AA. Both the intestinal lumen and the vascular tree were perfused with continuous fluid delivery using a Masterflex L/S variable speed roller pump at 1 ml/min. The intestine was infused with dH_2_O at 4 °C for 24 hours, 4% SDC at RT for 4 hours, and 2000 kU DNase-I in 1 M NaCl at RT for 3 hours. The process was repeated for up to four cycles. Following treatment, the constructs were preserved at 4 °C in PBS/AA. The samples were fixed in the same fashion as described the previously described samples; however, in this case they were not critical point dried. Tubular intestinal scaffolds, with both ends tied up in a “sausage” fashion and an average length of 2 cm, were soaked overnight in sterile 1 % penicillin/streptomycin (PS) in PBS.

### Imaging methods

All images were obtained with x-ray phase contrast (PC) computed tomography (CT), which has the capability to yield high contrast, high resolution, volumetric images without the need for destructive sample preparation. Unlike conventional CT (or microCT), where contrast arises from attenuation differences within the sample, contrast in PC-CT stems from the phase shift that x-rays suffer while they travel through matter^9^. This effect can be up to three orders of magnitude larger than attenuation^18^, which explains the highly increased contrast that has been observed with PC imaging for a broad range of biological specimens^17^,^19^,^20^,^21^,^22^,^23^,^24^,^25^.

#### Synchrotron-based PC-CT

**Figure 1:**
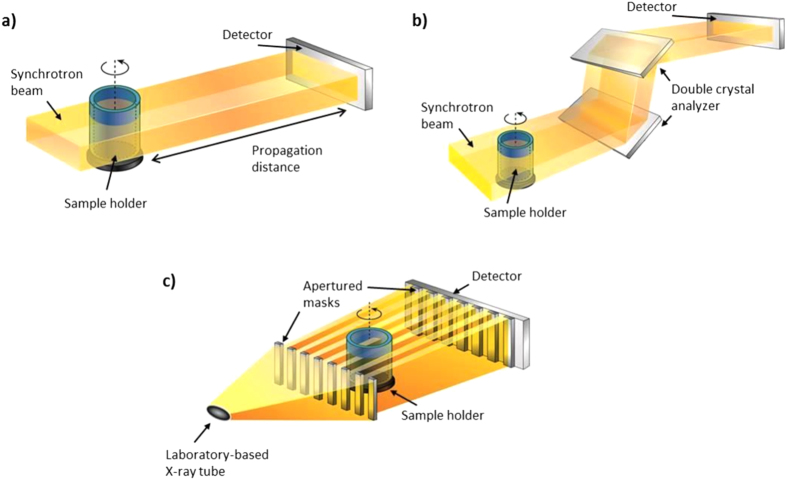
Schematics of the PC-CT methods used for the experiments: (a) “Propagation-based” PC-CT with synchrotron radiation, (b) “Analyzer-based” PC-CT with synchrotron radiation and (c) “Edge Illumination” PC-CT with a conventional x-ray tube.

Esophagus, lung and liver specimens were imaged using a “propagation-based” PC-CT setup implemented at the biomedical beamline (ID17) of the European Synchrotron Radiation Facility (ESRF; Grenoble, France). A schematic of the method is shown in Fig. 1(a); it comprises a (quasi) parallel synchrotron beam, a sample rotation stage and a pixelated detector. The key difference to a conventional radiographic setup is the enlarged distance between sample and detector which, coupled with a source of sufficient spatial coherence, enables interference fringes to develop as a consequence of the phase shift which x-rays suffer while they travel through the sample. The application of appropriate phase retrieval methods allows converting the fringes into phase-based area contrast^26^.

The source-to-sample and object-to-detector distances were approximately 150 m and 3.45 m, respectively. The source full width half maximum (FWHM) dimensions were 123 *μ*m horizontally and 24 *μ*m vertically. The beam was monochromatized by a fixed-exit Laue/Laue silicon (1,1,1) crystal to an energy of 26 keV (ΔE/E ≈ 0.02 %) and filtered using 0.8 mm of carbon and 3 mm of aluminium. The detector was the FReLoN CCD camera^27^ coupled to a 47 *μ*m thick GGG:Eu on GGG substrate scintillator, resulting in an effective pixel size of 3.5 × 3.5 *μ*m^2^. The CT scan involved the acquisition of 2000 equally spaced projections over a 360 degree rotation of the sample. The exposure time was 2 s per projection, and the total scan duration was approximately 1.5 h. The “single distance” phase retrieval algorithm by Paganin *et al.*^28^ was applied to the individual projections to convert the phase contrast fringes into phase-based area contrast, requiring an estimate of the phase-attenuation ratio (often termed “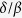-ratio”). The images were reconstructed with 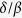-ratios in the range of 350–1000 (the precise values per image are given in the captions of the respective figures shown below); these were determined on the basis of what provided the best image quality. It should be noted that Paganin’s algorithm can potentially lead to a small sacrifice in spatial resolution, which could be overcome by the application of a more sophisticated phase retrieval method^29^. CT reconstruction was performed with FBP and in “half tomography” mode since the sample slightly moved out of the field of view during its rotation^30^. Images were processed using the ESRF in-house software PyHST, and analyzed and displayed with ImageJ^31^ and 3DSlicer.

Small intestine specimens were imaged with an “analyzer-based” PC-CT setup implemented at the SYRMEP (SYnchrotron Radiation for Medical Physics) beamline of the Elettra synchrotron (Trieste, Italy). A schematic of the method is shown in Fig. 1(b): it comprises a (quasi) parallel synchrotron beam, an object rotation stage, a double crystal analyzer and a pixelated detector. In contrast to “propagation-based” PC-CT, “analyzer-based” PC-CT is sensitive to x-ray refraction, which is directly proportional to the first derivative of the phase shift. X-ray refraction, i.e. microradian or sub-microradian deviations from the original propagation direction, is translated into image contrast via the crystal analyzer: obeying the laws of Bragg diffraction, the crystal analyzer modulates the beam intensity as a function of its incoming angle^32^. Consequently, the crystal causes the refracted parts of the beam to reach the detector with an increased or decreased intensity, depending on the direction and magnitude of the refraction angle. This manifests as “edge contrast” in the images, i.e. as dark and bright fringes on the contours and internal features of the sample.

The source-to-sample, sample-to-analyzer and analyzer-to-detector distances were approximately 23 m, 40 cm and 50 cm, respectively. The crystal analyzer was cut from two Si (1,1,1) crystals and tuned to the first half slope of its rocking curve^33^. The detector was a CCD camera (Photonic Science Ltd) with fibre optics taper coupled to Gd_2_O_2_S scintillating optics, resulting in an effective pixel size of 4.5 × 4.5 *μ*m^2^. The source FWHM dimensions were 135 *μ*m horizontally and 80 *μ*m vertically. The beam was monochromatized by a double crystal Si (1,1,1) monochromator to an energy of 17 keV (ΔE/E ≈ 0.2%). During the CT scan, 1440 equally spaced projections were acquired over 360°. The exposure time was 0.1 s per projection, and the total scan duration was 55 min. CT reconstruction was performed using FBP. Since no phase retrieval procedure has been applied, CT images showed attenuation in addition to “edge contrast” due to refraction within the sample. Moreover, some propagation-based phase contrast may have been present in the images due to uni-directional sensitivity of crystal analyzer and the non-negligible propagation distance (90 cm) between the sample and the detector. Images were processed using the PITRE software package^34^, and analyzed and displayed in ImageJ and OsiriX.

#### Laboratory-based PC-CT

One esophagus specimen was also imaged using an “edge illumination” PC-CT setup implemented with a commercially available x-ray tube in the radiation physics laboratory at University College London. Although this PC-CT implementation was initially developed at synchrotrons^35^,^36^, it does not rely on beam coherence^37^,^38^; hence, its working principle is not violated when implemented with x-ray tubes with relatively large focal spots. A schematic of the method is shown in Fig. 1(c): it comprises an incoherent (cone) beam, an object rotation stage, two apertured masks and a pixelated detector. The first apertured mask, positioned upstream of the sample, splits the incoming beam into an array of individual beamlets, with a lateral inter-beamlet distance sufficient to keep them physically separated. The second aperture mask, positioned in front of the detector, creates insensitive regions between adjacent pixel columns. When the first mask is slightly misaligned from the second, such that half of each beamlet falls onto an uncovered part of a pixel and the other half falls onto a covered one (“edge illumination” condition), sensitivity to refraction is achieved in addition to attenuation: x-rays that are deviated from their path cause either an increased or decreased intensity on the pixel, depending on the direction of deviation^37^. A dedicated phase retrieval procedure, requiring two input images acquired under opposing edge illumination conditions, enables a quantitative extraction of the refraction (i.e. differential phase) contrast^39^.

The source-to-sample and sample-to-detector distances were 1.6 m and 0.4 m, respectively. The setup features a Rigaku MicroMax 007 HF x-ray tube with rotating molybdenum target and a focal spot with a horizontal FWHM dimension of approximately 70 *μ*m. The tube was operated at 35 kV and 25 mA, corresponding to a broad spectrum with a mean energy of approximately 18 keV. The detector was the Hamamatsu C9732DK flat panel, a passive-pixel CMOS sensor with a matrix of 2400 × 2400 pixels and a pixel size of 50 × 50 *μ*m^2^. The periods of the first and second aperture masks (Creatv Microtech) were 79 *μ*m and 98 *μ*m, respectively, and their slit apertures were 23 *μ*m and 29 *μ*m wide, respectively. With these periods, every second pixel column was covered (“line-skipping” configuration), which reduces the effect of cross-talk between the pixels^40^. The field of view of the imaging system, as defined by the size of the masks, was 4.8 × 4.8 cm^2^. The CT scan involved acquiring 720 equally spaced projections over 360 degrees. At each rotation angle, two projections were acquired under opposing edge illumination conditions^39^. Moreover, at each angle, the sample was displaced ten times by 7.9 *μ*m (a tenth of the period in the first aperture mask), a projection was acquired at each displacement, and these projections were eventually recombined. This procedure, known as “dithering”, is used to increase the spatial resolution in the final image^41^,^42^, which in this case made the final resolution of the laboratory-based images comparable to that of images acquired at synchrotrons, where much smaller pixel sizes were used. It relies on the observation that in edge illumination PC-CT the spatial resolution is independent from the pixel size^41^, an important feature that has already been exploited for hard x-ray microscopy implementations^43^. Each projection was acquired with an exposure time of 1.2 s. The total live exposure time of the sample was 4.8 h, however the entire scanning procedure took approximately 19 h due to the fact that the imaging setup had not been optimized for speed for this feasibility study and excessive time was spent on motor movements, detector read-out and flat field acquisitions. In an optimized setup, this would be significantly shortened. It terms of the total exposure time, it should be noted that for this sample a resolution much higher than that dictated by the pixel was required, and hence 10 dithering steps were used, implying a factor of 10 in total exposure. If the pixel level resolution is sufficient, a significantly reduced exposure time can be expected. We would also like to highlight that the total exposure could be reduced by an additional factor of two through the use of a recently published phase retrieval procedure which is based on the acquisition of one image at each angle only^44^. Following phase retrieval^39^, CT reconstruction was performed with FBP. A specialized filter function was used to account for the differential nature of edge illumination images, converting the “edge contrast” that is due to refraction into phase-based area contrast^45^. A MATLAB routine was written to perform the image processing. Images were analyzed and displayed using ImageJ and 3DSlicer.

## Results

Results are presented and discussed individually for each organ (esophagus, lung, liver and small intestine).

### Esophagus

**Figure 2:**
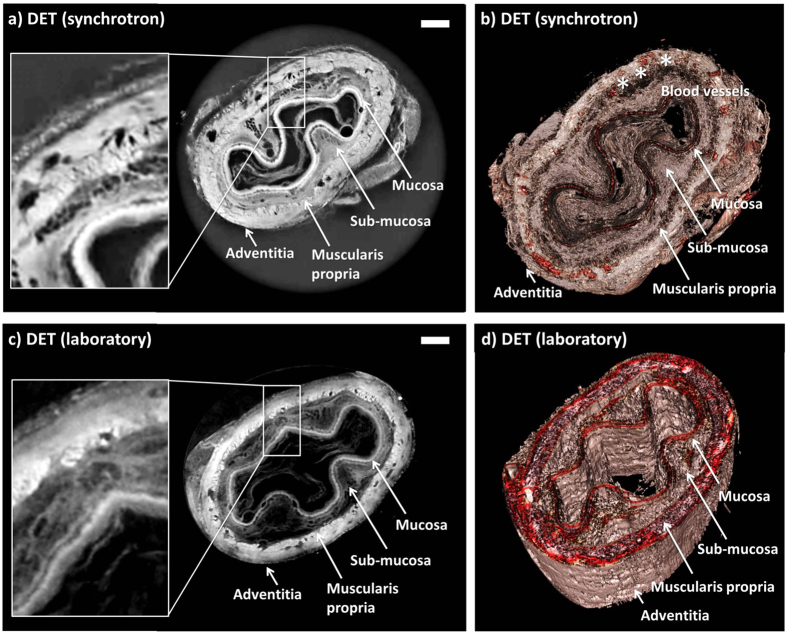
Images of DET-decellularized rabbit esophagi acquired with synchrotron-based PC-CT (reconstructed with *δ*/*β* = 350) [(a,b)] and laboratory-based PC-CT [(c,d)]. The images show transverse cross sections of the specimens [(**a**,**c**)], and three-dimensional views [(**b**,**d**)]. All scale bars represent 500 *μ*m.

Rabbit esophagi were successfully decellularized after three cycles of DET, as evident by histology and DNA quantification (data not shown). Images of an esophageal scaffold acquired with synchrotron-based PC-CT are shown in Fig. 2(a,b) in form of a transverse cross section through the reconstructed volume and a three-dimensional rendering, respectively. The corresponding images acquired with laboratory-based PC-CT are shown in Fig. 2(c,d). All images revealed intact scaffold microarchitecture, allowing the identification of all layers of the native tissue, namely: mucosa, sub-mucosa, muscularis propria (MP), and adventitia. Intact blood vessels were detected in the submucosa and a clear demarcation was seen between the inner circular and outer longitudinal layers of the MP. Owing to strong image contrast, the layers were easily identified and distinguished, although all of them are composed of soft tissue with extremely similar x-ray attenuation properties and would be indistinguishable to conventional microCT. The mucosal-submucosal separation (MSS), a surrogate marker for decellularization-associated damage, could be detected across the entire circumference of a long segment of the scaffold and shown to be minimal. The three-dimensional renderings in Fig. 2(b,d) allow a fully volumetric analysis of the MSS, which is an advantage over histology and SEM. It should be noted that the image quality in the synchrotron-based and laboratory-based images is comparable. This is remarkable, considering that the former were acquired with highly brilliant and coherent synchrotron radiation, while the latter were acquired with a commercially available x-ray tube.

### Lung

**Figure 3:**
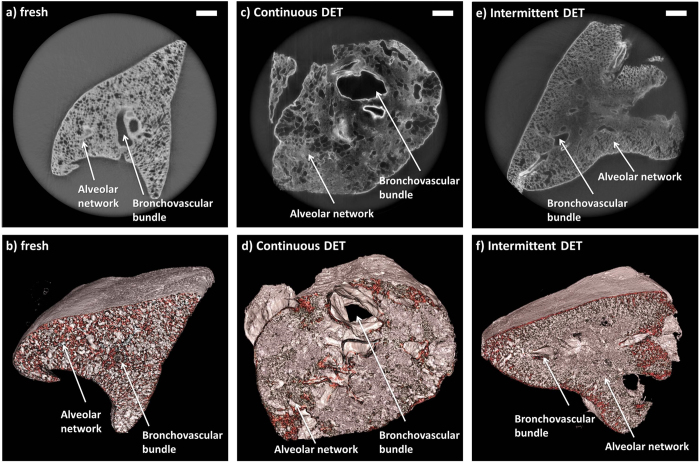
Images acquired with the synchrotron-based PC-CT showing a fresh rat lung (reconstructed with *δ*/*β* = 550) [(a,b)] and rat lung specimens that were decellularized via continuous DET (reconstructed with *δ*/*β* = 1000) [(c,d)] and intermittent DET (reconstructed with *δ*/*β* = 1000) [(e,f)]. The images show transverse cross sections through the samples [(**a**,**c**,**e**)], and three-dimensional views [(**b**,**d**,**f**)]. All scale bars represent 500 *μ*m.

Rat lungs were successfully decellurarized as previously described^16^. Previous work was aimed at establishing whether DET with continuous or intermittent inflation would maintain microarchitecture best, and the intermittent inflation approach was found to be superior, as shown by SEM and functional analyses. Synchrotron-based PC-CT imaging of the lung specimens supported this finding. The images of a control fresh tissue, shown Fig. 3(a,b) in the form of a transverse cross section and a three-dimensional rendering, respectively, exhibit a centrally placed bronchovascular bundle and a peripheral tightly packed alveolar network. The images of a lung specimen that was decellularized via continuous DET [Fig. 3(c,d)] show an ACM in which the bronchovascular bundle was enlarged with thinned out, compressed walls and ambiguous architecture. This can be especially appreciated in the three-dimensional rendering of the scaffold [Fig. 3(d)]. The changes in the alveolar network were also pronounced, either showing atectactic changes, secondary to compression or enlargement due to rupture of the alveolar-capillary membrane and the union of multiple alveoli. In comparison to this, it could be observed that intermittent DET produced an ACM in which both the bronchovascular bundle and the alveolar network were completely preserved [Fig. 3(e,f)].

### Liver

**Figure 4:**
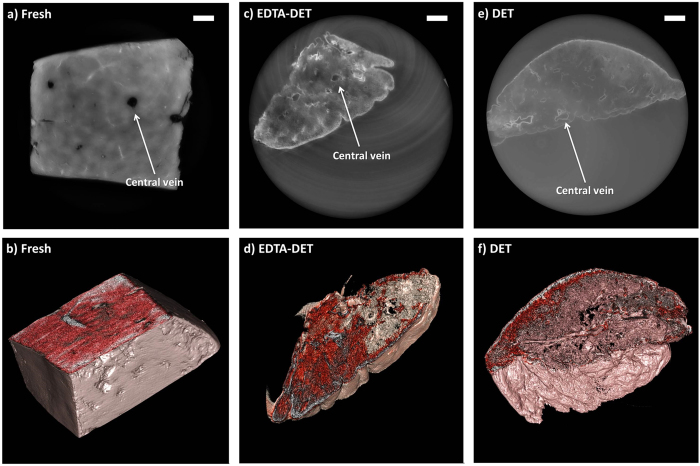
Images acquired with synchrotron-based PC-CT showing a fresh rat liver (reconstructed with *δ*/*β* = 350) [(a,b)] and rat liver specimens that were decellularized via EDTA-DET (reconstructed with *δ*/*β* = 600) [(c,d)] and DET (reconstructed with *δ*/*β* = 800) [(e,f)]. The images show transverse cross sections through the samples [(**a**,**c**,**e**)], and three-dimensional views [(**b**,**d**,**f**)]. All scale bars represent 500 *μ*m.

A previous study (Maghsoudlou *et al.*, submitted) was aimed at establishing the effectiveness of DET and EDTA-DET protocols for the generation of ACMs from rat liver. The outcome was that DET yields a microarchitecture that is better preserved compared to EDTA-DET. This observation is confirmed by synchrotron-based PC-CT images of the liver specimens [Fig. 4]. The fresh liver specimen is composed of a tight tissue structure, revealing hexagonal lobules and several branches of the central and portal veins [Fig. 4(a,b)]. The main vessels can be seen to be similarly preserved in tissues decellularized with both EDTA-DET [Fig. 4(c,d)] and DET [Fig. 4(e,f)]. The increased intensity in the EDTA-DET scaffold [Fig. 4(c)] compared to the DET scaffold [Fig. 4(e)] suggests that the EDTA-DET protocol produces a denser tissue, as was the main finding in the published study. In addition, the EDTA-DET scaffold showed areas of both hyper- and hypo-intensity, while the DET scaffold had homogeneous features. This finding could not be appreciated by SEM analysis.

### Small intestine

**Figure 5:**
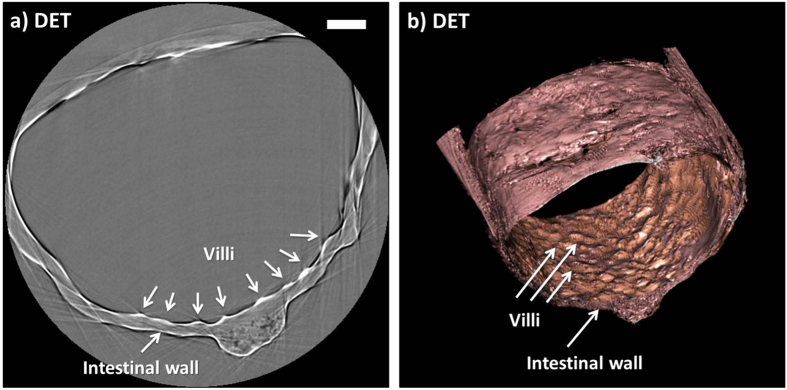
Images acquired with synchrotron-based PC-CT showing a rat small intestine that was decellularized via DET: (a) transverse cross section, (b) three-dimensional view. All scale bars represent 500 *μ*m.

Images of the rat small intestine acellular matrix acquired with synchrotron-based PC-CT are shown in Fig. 5: a transverse cross section through the cylindrical specimen and a three-dimensional rendering of the imaged section of the organ can be seen in Fig. 5(a,b), respectively. To demonstrate the flexibility of the PC-CT approach to the imaging of decellularized scaffolds, images were acquired with a different implementation of PC-CT (“analyzer-based” instead of “propagation-based” PC-CT^9^). Furthermore, the “edge contrast” that is due to refraction within the sample was not converted into phase-based area contrast, which explains why the contrast in Fig. 5(a) is strongest at the interfaces, where it manifests as bright and dark fringes. Since the colour scheme has been selected as to create the impression of area contrast, this is not as obvious in the volume rendering. In addition, the sample preparation was slightly modified for this experiment: the specimen was hydrated instead of critical point dried.

Previous SEM imaging has shown that DET preserves the villi/crypt microstructure in small intestine specimens^6^. The PC-CT images shown in Fig. 5 confirm this observation, in the sense that the presence of villi in the scaffold can clearly be appreciated. However, crypts, i.e. the small cavities between the villi, are not visible. This is due to insufficient spatial resolution of the used detector: while the villi in a mouse small intestine are typically of sizes of tens of micrometres, the crypts are as small as a few micrometres, i.e. too small to be resolved by the detector pixel used in this case. To investigate the capability of PC-CT to resolve intestinal crypts, the imaging of the small intestine ACM will be repeated with higher resolution detector as part of future work.

## Discussion

A range of fresh and decellularized organs (esophagus, lung, liver, small intestine) originating from rabbits and rats was imaged with synchrotron and laboratory implementations of PC-CT. All images were of very high quality, especially with regards to contrast and detail visibility, to a degree that the findings obtained previously with histology and SEM could be confirmed^6^,^16^. Anatomical features such as the mucosal-submucosal separation in the esophageal scaffold and the heterogeneity of the EDTA-DET liver scaffold could be identified. Native scaffold microarchitecture was clearly visible in all images, allowing the validation of the decellularization methods used. The fact that this has been demonstrated for a wide range of tissue types and for different implementations of PC-CT demonstrates the versatility of the method and its reliability in a variety of TE areas. Most importantly, the fact that an image quality comparable to that of synchrotron-based PC-CT was obtained in a standard laboratory using exclusively conventional x-ray equipment indicates that not only does PC-CT have the capability to replace histology and SEM for this range of applications, but also that the imaging could be performed inside TE research laboratories, enabling a high-throughput and wide uptake.

The key difference between PC-CT and the gold-standard methods currently used by the TE community to assess microstructure preservation in scaffolds (SEM and histology) is that PC-CT is non-destructive, which makes it a long-sought solution for imaging scaffolds prior to transplantation. This means that PC-CT could ultimately become a tool for routine quality control in regenerative medicine, as well as potentially be used for monitoring scaffold behaviour and functionality *in vivo* after transplantation into live organisms, e.g. mice. For this application, it is important that the delivered radiation dose is carefully monitored and kept below acceptable limits. In this context, it should be noted that PC imaging has been shown in several instances to have low-dose capabilities which are compatible with pre-clinical and clinical standards^38^,^46^,^47^.

Finally, the intrinsic volumetric nature of PC-CT provides a precise understanding of the scaffold microarchitecture in three dimensions. This is required to develop the knowledge necessary to fabricate synthetic scaffolds that could eventually replace ACMs of intricate organs such as those investigated in this study. Synthetic scaffolds have the advantage that they are man-made, can be produced on a semi-industrial scale and, hence, made widely available to the population.

## Additional Information

**How to cite this article**: Hagen, C. K. *et al.* High contrast microstructural visualization of natural acellular matrices by means of phase-based x-ray tomography. *Sci. Rep.* **5**, 18156; doi: 10.1038/srep18156 (2015).
